# Retraction: Oral Administration of GW788388, an Inhibitor of Transforming Growth Factor Beta Signaling, Prevents Heart Fibrosis in Chagas Disease

**DOI:** 10.1371/journal.pntd.0012360

**Published:** 2024-07-23

**Authors:** The PLOS Neglected Tropical Diseases Editors

Following publication of this article [1] concerns were raised regarding Figs 5 and 6. Upon editorial follow-up, the corresponding author indicated there were errors in figure preparation. Specifically:

- In Fig 5E, the incorrect lanes were selected for inclusion in the lower GAPDH panel such that the GAPDH bands shown are not the correctly aligned loading controls for the collagen type I panel.
- The GAPDH panel in Fig 6F is incorrect: lanes 1–3 are duplicates of lanes 7–9 of the lower GAPDH panel in Fig 5E when rotated 180 degrees and horizontally flipped, and lanes 4–9 are duplicates of lanes 1–6 of the upper GAPDH panel in Fig 5E when rotated 180 degrees.

The authors stated that the quantitative analysis of the western blots was performed using the correct images despite this error in the published article, and they provided normalized quantitative data. Given the nature of the issues and the absence of raw densitometry data, the *PLOS Neglected Tropical Diseases* Editors remain concerned about the reliability of these results.

The corresponding author provided underlying blot data for Figs 5E and 6F. In images of the Fig 6F blots taken after 10 second and 1 minute exposures, the signal did not appear to increase consistently across the blot after the longer exposure. In addition, the published Collagen Type 1 panel in Fig 6F appears to contain lanes from both the 10 second and 1 minute exposures. Lanes 1–4 appear to be from the 10 second exposure and lanes 5–9 appear to be from the 1 minute exposure. These issues raise concerns about the reliability of the densitometry data based on these images.

In light of the unresolved concerns that question the reliability of these data, the *PLOS Neglected Tropical Diseases* Editors retract this article.

TCAJ, EMdS, GMdO, WMD, JJF, SB, and MCW did not agree with the retraction. FLdO either did not respond directly or could not be reached.
